# Frailty transitions and prevalence in an ageing population: longitudinal analysis of primary care data from an open cohort of adults aged 50 and over in England, 2006–2017

**DOI:** 10.1093/ageing/afad058

**Published:** 2023-05-02

**Authors:** Bronagh Walsh, Carole Fogg, Scott Harris, Paul Roderick, Simon de Lusignan, Tracey England, Andrew Clegg, Sally Brailsford, Simon D S Fraser

**Affiliations:** School of Health Sciences, Faculty of Environmental and Life Sciences, University of Southampton, Southampton, UK; School of Health Sciences, Faculty of Environmental and Life Sciences, University of Southampton, Southampton, UK; School of Primary Care, Population Science & Medical Education, Faculty of Medicine, University of Southampton, Southampton, UK; School of Primary Care, Population Science & Medical Education, Faculty of Medicine, University of Southampton, Southampton, UK; Nuffield Department of Primary Care Health Sciences, University of Oxford, Oxford, UK; School of Health Sciences, Faculty of Environmental and Life Sciences, University of Southampton, Southampton, UK; Academic Unit of Elderly Care and Rehabilitation, University of Leeds, Leeds, UK; Southampton Business School, University of Southampton, Southampton, UK; School of Primary Care, Population Science & Medical Education, Faculty of Medicine, University of Southampton, Southampton, UK

**Keywords:** frailty, incidence, prevalence, transitions, ageing population, older people

## Abstract

**Introduction:**

frailty is common in older adults and is associated with increased health and social care use. Longitudinal information is needed on population-level incidence, prevalence and frailty progression to plan services to meet future population needs.

**Methods:**

retrospective open cohort study using electronic health records of adults aged ≥50 from primary care in England, 2006–2017. Frailty was calculated annually using the electronic Frailty Index (eFI). Multistate models estimated transition rates between each frailty category, adjusting for sociodemographic characteristics. Prevalence overall for each eFI category (fit, mild, moderate and severe) was calculated.

**Results:**

the cohort included 2,171,497 patients and 15,514,734 person-years. Frailty prevalence increased from 26.5 (2006) to 38.9% (2017). The average age of frailty onset was 69; however, 10.8% of people aged 50–64 were already frail in 2006. Estimated transitions from fit to any level of frailty were 48/1,000 person-years aged 50–64, 130/1,000 person-years aged 65–74, 214/1,000 person-years aged 75–84 and 380/1,000 person-years aged ≥ 85. Transitions were independently associated with older age, higher deprivation, female sex, Asian ethnicity and urban dwelling. Mean time spent in each frailty category decreased with age, with the longest period spent in severe frailty at all ages.

**Conclusions:**

frailty is prevalent in adults aged ≥50 and time spent in successive frailty states is longer as frailty progresses, resulting in extended healthcare burden. Larger population numbers and fewer transitions in adults aged 50–64 present an opportunity for earlier identification and intervention. A large increase in frailty over 12 years highlights the urgency of informed service planning in ageing populations.

## Key Points

- Frailty is already present in the population before age 65.
- Longer times spent in moderate and severe frailty suggest extended burden of disease.
- Frailty progresses more rapidly with increasing age, resulting in high prevalence.
- Frailty transitions are associated with increasing age, higher deprivation, female sex, Asian ethnicity and urban dwelling.
- Strategies to reduce the burden of frailty need to consider health inequalities.

## Introduction

As populations age, healthcare systems must identify ways of meeting changing needs while managing growing demand [1]. This is particularly important for older people living with frailty, a condition characterised by reduced physiological reserve and vulnerability to stressor events [2, 3]. Frailty is associated with increased mortality and health and social care service use, dependency and reduced quality of life [4–9]. Risk factors for frailty include female sex, deprivation, ethnicity and multiple morbidities [10, 11]. Consensus guidelines on the management of frailty [4, 5, 12, 13] recommend the identification of frailty and targeted clinical management to enhance the quality and appropriateness of care. Planning and resourcing such interventions require high-quality population-level data on expected trends and transitions in frailty. Such data would also aid population-level strategies for frailty prevention and slowing frailty progression, key in reducing future burden on patients and care services [14]. The relationship between transitions in frailty indices and outcomes has been explored using multistate models [14–16]. However, these models were based on prospective cohorts of moderate size, included a limited number of covariates and had few follow-up time points. There is a need for further information on the progression of frailty in the general population and the prediction of transitions to more severe frailty states over shorter time frames to adequately inform service development and public health interventions. Moreover, little information is available on frailty transitions in adults aged 50–64 to facilitate earlier intervention.

It is estimated that 1.8 million people in the UK aged ≥60 were living with frailty in 2016 [6], with prevalence rising from 6.5% in people aged 60–69 to 65% of those aged ≥90 [17]. International estimates vary widely, from 3.9 (China) to 51.4% (Cuba), with a pooled prevalence of 17.4% [18] in low- and middle-income countries and 12% in pooled data from high-income countries [19]. Frailty incidence estimates vary from 12 to 204 cases per 1,000 person-years at risk (PYAR), with a pooled incidence of 43.4/1,000 PYAR [20]. A systematic review of studies using phenotypic assessment of frailty reported 29.1% of people progressed to a worsened frailty state over a mean period of3.9 year, with 4.5% moving from robust to frail and 18.2% from pre-frail to frail [21]. However, as with incidence and prevalence, the studies were diverse in design, including generally <5,000 participants and around 4 years of follow-up, and used measures and cut-off scores not directly comparable to frailty index (FI) scores calculated using electronic health records (EHR). Few studies use frailty indices that could feasibly be applied to routine EHR data, essential for large-scale population-level analyses needed for service planning. Heterogeneity in age ranges, follow-up duration and differing frailty measures make meaningful synthesis challenging. Evidence from validation of the electronic Frailty Index (eFI), a cumulative measure of 36 long-term conditions, disabilities, clinical signs and symptoms and abnormal test values, developed using EHRs in England [22], suggests progression of frailty accelerates over time [23]. A Dutch study using a 32-item frailty index [24] described a doubling in deficits over an average of 12.6 years [25].

Further evidence on frailty progression within the ageing population is needed from large-scale population studies designed for this purpose. This study addresses these evidence gaps using longitudinal analyses to explore expected transitions within the older population in primary care. This work builds on what is known about individual risk of frailty onset and progression.

## Aims

This study is part of a larger programme of work, which aims to explore and predict trends in onset, prevalence and progression of frailty, and the dynamics of frailty-related healthcare demand, outcomes and costs in the ageing population. This paper presents results relating to prevalence and transitions into and between frailty states in people aged ≥50 over 12 years. Transition probabilities reported here will inform the development of a predictive simulation model for use in estimating service demand and outcomes in the ageing population.

## Methods

### Study design

Retrospective open cohort study using EHR from the Royal College of General Practitioners (RCGP) Research and Surveillance Centre (RSC) sentinel network, which at the time of the study collated routine primary care data from >500 GP practices in England and is nationally representative [26].

### Population and sample size

Primary care patients, aged 50 years and above, registered at General Practitioner (GP) practices contributing to the RCGP RSC databank between 2006 and 2017 were eligible. The sample size was maximised to allow robust analysis by age and other sub-groups of interest. This was achieved by using retrospective data from the most recent complete year at the point of data extraction and all preceding years with the availability of study variables, a total of 12 years. The open cohort design enabled the addition of eligible patients who turned 50 or moved to a participating practice and were present on 1st January of a calendar year during the study period. Patients left the cohort by leaving participating practices or death. The cohort comprised 2,177,656 patients from 419 GP practices across England (previously described in [27]). Patient follow-up data were removed where the data were discrepant with ONS deaths (6,159 patients and 38,212 follow-up years). A total of 2,171,497 patients were analysed, with 1,104,135 patients in 2006 rising to 1,489,495 in 2017. Over the study period, 1,067,362 patients entered, 355,889 died (16.4%) and 411,378 (18.9%) de-registered from RCGP practices.

### Primary outcome measure

The primary outcome of frailty was measured by calculating an eFI score [22] from electronic primary care health records on January 1st for each calendar year for each participant. The eFI score was calculated by automatically searching the primary care record for the presence of Read codes relating to the 36 deficits [22]. The score is calculated as the number of deficits ever recorded/36. A frailty category was assigned according to the eFI score cut-offs: fit (0–0.12), mild (0.13–0.24), moderate (0.25–0.36) and severe (>0.36), in line with FI categories described in the literature and reflecting cut-offs used in practice [22, 28, 29].

### Additional measures

The RCGP RSC dataset included: age category (50–64, 65–74, 75–84 and 85+); sex; ethnicity; 2015 indices of multiple deprivation (IMD) quintiles [30]; income deprivation affecting older people index (IDAOPI, the proportion of people aged ≥60 who experience income deprivation) quintiles [31]; record of residential care during the follow-up period; rural/urban location (according to the UK Rural Urban Classification https://www.gov.uk/government/collections/rural-urban-classification); date of cohort entry; date of cohort exit (leaving a contributing practice or death). Age groups were chosen to reflect those commonly used in the frailty literature, allowing for exploration of the middle-aged to younger old and presentation of findings in groups relevant to service planners. The dataset was supplemented by linked data from the UK Office for National Statistics (deaths occurring within any calendar year for which a patient was present in the cohort on January 1st) and NHS Digital (ethnicity data). Ethnicity data were derived from both primary and secondary care data to minimise missing data according to the 16 categories in the NHS data dictionary (https://www.datadictionary.nhs.uk/data_elements/ethnic_category.html). To enable suitably sized categories for analysis, the categories were further aggregated into Asian, Black, Mixed/Other and White, where known.

### Statistical analysis

Frailty prevalence was calculated as per the eFI on January 1st for each calendar year, including all persons present in the cohort at that date, and stratified by age group and severity. Frailty incidence rates (new onset frailty of any severity) were calculated per 1,000 person-years across the 12-year study period.

Continuous time multi-state Markov (MSM) models were used to estimate transition rates between states and identify determinants of frailty progression [32–34]. Variables used in the model reflected non-modifiable, population factors associated with frailty, including socioeconomic and demographic variables, relevant and available to service planners. During each year of follow-up, the frailty category for each individual was treated as their current state, with a final absorbing state of death from any cause. The assumptions of the fitted models were that; exact transition times were not observed; multiple transitions could occur between observation points, with patients passing through intermediate states; date of death was assumed to be recorded exactly (Appendix 1). The eFI score is calculated from accumulated diagnoses and problems recorded in general practice; although it is possible to measure improvements in frailty status, in practice, conditions are unlikely to be removed from the record and reversals in score were uncommon in our dataset. Reverse transitions were therefore excluded from the model design, although reversals due to polypharmacy were noted in 3.9% of patients; these were imputed to the most recent higher frailty category.

An initial unadjusted MSM model was fitted to estimate average annual transition probabilities. Multivariable models were then fitted to assess the impact of the key sociodemographic variables of age group, sex, ethnicity (Black, Asian, White or other and Unknown), deprivation (grouped as the two most deprived quintiles versus the 3 least deprived) and rural/urban location on these transitions, in a forward selection process. The Akaike information criterion and likelihood ratio test were used to compare and choose between models. SAS version 9.4, R version 4.2.0 and Stata version 16.0 software were used for data manipulation and statistical analyses. The R msm package version 1.6.9 was used for the MSM modelling [33]. *P* values <0.05 were considered statistically significant and estimates are presented with 95% confidence intervals (CIs) where appropriate.

### Ethics

The study was approved by the University of Southampton Research Ethics Committee (ref 46313) on 6 February 2019, the RCGP RSC Information Governance Panel on 24 January 2019 and NHS Digital’s Data Access Request Service (DARS) Independent Group Advising on the Release of Data (IGARD) panel on 19 April 2021.

## Results

The cohort comprised 2,171,497 patients, contributing 15,514,734 person-years of data, with a median follow-up of 7 years (interquartile range 7 years).

The average age of onset for frailty (any category) for patients who were fit at cohort entry was 69 years (Standard deviation (SD) 10 years). The overall frailty incidence rate was 47.1 cases per 1,000 person-years (95% CI 47.0–47.2). Crude incidence was higher in older age groups, female sex, Asian ethnicity, more deprived quintiles and people living in urban areas (Table 1). Incidence rates were 31.8/1,000 for the 50–65 year age group, rising to 158.5/1,000 for the oldest. Rates remained stable in the 50–64 age group due to the open nature of the cohort, but gradually decreased in older age groups as prevalence increased and fewer non-frail people were present (Appendix 2).

**Table 1:** Crude incidence rates of frailty by patient characteristics from 2006 to 2017

**Characteristic**	**Category**	***n* (%)** ^**a**^	**Number fit at cohort entry (%)** ^**a**^	**Incidence rate per 1,000 person-years at risk (95% CI)**
		*N* = 2,171,497	*N* = 1,700,724	
**Age at cohort entry**	50–64	1,412,823 (65.1%)	1,272,762 (74.8%)	31.8 (31.7–32.0)
	65–74	384,640 (17.1%)	272,232 (16.0%)	85.2 (84.7–85.6)
	75–84	257,276 (11.9%)	119,597 (7.0%)	136.9 (135.9–137.9)
	≥85	116,758 (5.4%)	36,133 (2.1%)	158.5 (156.2–160.8)
				
**Sex**	Male	1,040,906 (48.0%)	855,015 (50.3%)	42.2 (42.0–42.4)
	Female	1,130,591 (52.1%)	845,709 (49.7%)	52.1 (51.9–52.3)
				
**Ethnicity** ^**b**^	Asian	73,932 (3.8%)	56,482 (3.8%)	57.3 (56.4–58.2)
	Black	40,122 (2.1%)	32,761 (2.2%)	49.1 (48.0–50.3)
	Mixed/Other	24,235 (1.3%)	20,292 (1.4%)	42.8 (41.6–44.1)
	White	1,807,038 (92.9%)	1,392,050 (92.7%)	50.9 (50.7–51.0)
				
**Location**	Urban	1,684,020 (77.6%)	1,311,431 (77.1%)	47.8 (47.6–47.9)
	Rural	487,477 (22.5%)	389,293 (22.9%)	45.0 (44.8–45.3)
				
**Residential care** ^**c**^	Yes	16,647 (0.77%)	3,317 (0.20%)	307.8 (298.4–317.5)
	No	2,154,850 (99.2%)	1,697,407 (98.8%)	46.8 (46.6–46.9)
				
**IMD**	Most deprived	290,760 (13.4%)	212,867 (12.5%)	57.9 (57.4–58.3)
	2nd quintile	341,323 (15.7%)	261,520 (15.4%)	51.1 (50.8–51.5)
	3rd quintile	439,069 (20.2%)	343,472 (20.2%)	47.6 (47.3–47.9)
	4th quintile	524,849 (24.2%)	417,448 (24.5%)	44.8 (44.5–45.0)
	Least deprived	575,496 (26.5%)	465,417 (27.4%)	42.7 (42.4–42.9)
				
**IDAOPI**	Most deprived	298,519 (13.8%)	220,689 (13.0%)	57.5 (57.1–58.0)
	2nd quintile	337,977 (15.6%)	254,043 (14.9%)	52.3 (51.9–52.6)
	3rd quintile	427,344 (19.7%)	331,038 (19.5%)	48.6 (48.3–48.9)
	4th quintile	520,409 (24.0%)	413,922 (24.3%)	44.9 (44.7–45.2)
	Least deprived	587,248 (27.0%)	481,032 (28.3%)	41.6 (41.4–41.9)
				

^a^% of patients with a known value for the characteristic.

^b^About 226,170 (10.4%) patients with missing values.

^c^Defined as people in receipt of residential care at some point during their follow-up period.

The analysis demonstrated at least one transition between frailty categories in 32.7% (*n* = 709,377) of the cohort over a median follow-up of 7 years. The average age of transition from fit to mild was 69 years (SD 10 years), fit/mild to moderate was 77 years (SD 10 years) and any category to severe was 81 years (SD 9 years).

**Figure 1:**
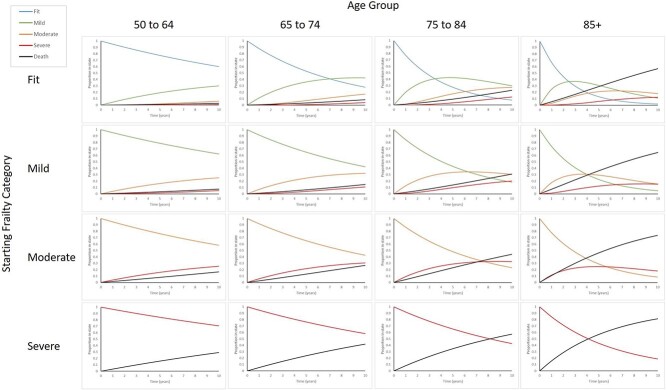
Proportion transitioning into different frailty categories (states), by starting frailty categories and age groups (fully adjusted model). Cohort age structure changed slightly over time, with 524,936 (47.5%) aged ≥65 in 2006 and 735,936 (49.4%) in 2017, for age 85+ this was 68,332 (6.2%) and 102,949 (6.9%), respectively. Over the same period, overall prevalence of frailty increased from 26.5 (95% CI 26.4–26.6) to 38.9% (95% CI 38.8–39.0). Frailty was already present in the 50–64 group, rising from 10.8% in 2006 to 19.6% in 2017 and prevalence increased with age (Appendix 3). Prevalence increased in all frailty categories, with the greatest proportion seen in mild and moderate frailty in all age groups (Figure 2). Total numbers with frailty increased from 292,751 to 579,828, with the greatest increase in numbers seen in the 65–74 age group and mild frailty category.

The multi-state model included, in order of decreasing impact, the following statistically significant predictors of frailty transitions: age group, deprivation, sex, ethnicity and urban/rural location. The number of people transitioning to a higher frailty category per 1,000 in one year was greater with each increase in age group (Table 2). The mean time spent within each frailty state decreased with age (Table 3), indicating that frailty progresses more rapidly with older age (Figure 1), with the longest period in severe frailty at all ages (Table 3).

**Table 2:** Hazard ratios and 95% CIs of the key sociodemographic variables for transitioning into different frailty states (fully adjusted model)

		Hazard ratio (95% CI) for the listed transition						
Sociodemographic variable		Fit to mild	Mild to moderate	Moderate to severe	Fit to death	Mild to death	Moderate to death	Severe to death
**Age group**	50—64	1	1	1	1	1	1	1
	65–74	2.44 (2.42–2.45)	1.80 (1.78–1.83)	1.55 (1.51–1.60)	2.65 (2.60–2.71)	1.81 (1.76–1.85)	1.64 (1.58–1.71)	1.56 (1.46–1.67)
	75–84	4.90 (4.86–4.93)	3.52 (3.48–3.56)	2.60 (2.53–2.67)	7.16 (7.00–7.31)	3.84 (3.75–3.92)	2.93 (2.83–3.04)	2.45 (2.30–2.61)
	85+	7.68 (7.59–7.77)	5.50 (5.43–5.57)	3.57 (3.48–3.67)	27.53 (26.89–28.19)	11.61 (11.37–11.87)	6.98 (6.73–7.23)	4.79 (4.50–5.11)
**Deprivation**	Least deprived (3–5)	1	1	1	1	1	1	1
	Most deprived (1–2)	1.25 (1.25–1.26)	1.23 (1.22–1.24)	1.18 (1.16–1.19)	1.49 (1.46–1.52)	1.36 (1.34–1.38)	1.17 (1.15–1.19)	1.07 (1.05–1.09)
**Sex**	Male	1	1	1	1	1	1	1
	Female	1.13 (1.13–1.14)	1.03 (1.02–1.03)	1.02 (1.01–1.04)	0.75 (0.74–0.77)	0.71 (0.70–0.72)	0.71 (0.70–0.72)	0.71 (0.70–0.72)
**Ethnicity**	White/Other	1	1	1	1	1	1	1
	Asian	1.28 (1.26–1.30)	1.15 (1.13–1.18)	1.01 (0.97–1.04)	0.54 (0.50–0.58)	0.54 (0.51–0.57)	0.65 (0.61–0.68)	0.74 (0.70–0.79)
	Black	1.04 (1.02–1.07)	0.97 (0.94–1.0)	0.94 (0.89–1.00)	0.74 (0.68–0.80)	0.67 (0.62–0.72)	0.77 (0.71–0.83)	0.73 (0.66–0.81)
	Not stated	0.21 (0.21–0.22)	0.49 (0.48–0.51)	0.59 (0.55–0.64)	1.28 (1.25–1.31)	3.28 (3.21–3.35)	3.46 (3.36–3.56)	3.01 (2.87–3.15)
**Urban status**	Rural	1	1	1	1	1	1	1
	Urban	1.06 (1.05–1.06)	1.06 (1.05–1.07)	1.07 (1.05–1.09)	1.00 (0.98–1.02)	0.98 (0.96–0.99)	0.97 (0.95–0.99)	0.96 (0.94–0.98)

**Table 3:** Number of people transitioning between frailty category per 1,000 person-years stratified by age group, adjusted for sex, deprivation, ethnicity and location

			**Incidence of transition to a different frailty category after 1 year per 1,000 person-years at risk (PYAR)**			**Died (per 1,000 PYAR)**
**Frailty category at the beginning of the year by age group**	**Number per 1,000 remaining in category**	**Time in category (years) mean (SEM)**	**Mild**	**Moderate**	**Severe**	
**Fit**						
50–64	950	19.62 (0.171)	47	1	0	2
65–74	880	7.82 (0.069)	111	4	0	5
75–84	772	3.87 (0.035)	198	15	1	14
85+	666	2.46 (0.025)	250	29	1	53
**Mild**						
50–64	954	21.01 (0.237)	–	40	1	6
65–74	918	11.63 (0.130)	–	70	2	11
75–84	844	5.91 (0.066)	–	126	7	24
85+	743	3.36 (0.039)	–	173	13	72
**Moderate**			–			
50–64	947	18.47 (0.342)	–	–	38	15
65–74	918	11.72 (0.199)	–	–	57	25
75–84	864	6.86 (0.114)	–	–	91	45
85+	781	4.05 (0.069)	–	–	113	106
**Severe**						
50–64	966	28.91 (1.299)	–	–	–	34
65–74	948	18.53 (0.665)	–	–	–	53
75–84	918	11.74 (0.396)	–	–	–	82
85+	845	5.94 (0.200)	–	–	–	155

Note: row totals may be less than or exceed 1,000 due to rounding; SEM = standard error for the mean.

**Figure 2:**
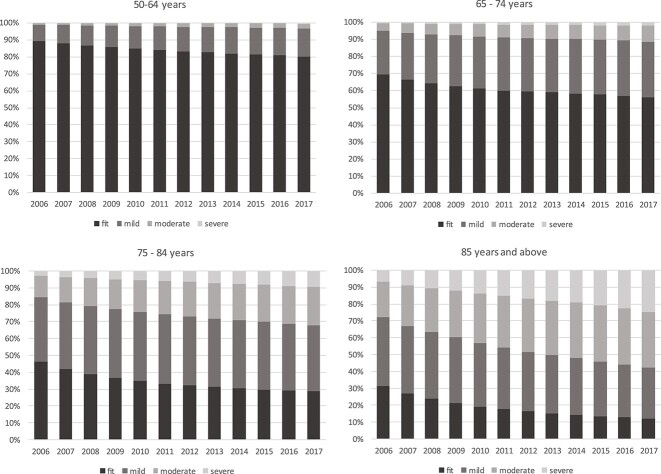
Prevalence of frailty categories 2006–2017 by age group.

## Discussion

A key strength of this study was its use of a large, population-level dataset with a long period of follow-up which allowed multistate modelling to describe and predict transitions between frailty categories within an ageing population over time. This has allowed precise estimates of transitions and prevalence at whole population level and within sub-groups of interest.

Our analysis has provided new evidence on frailty incidence, prevalence and transitions in an ageing population. This analysis suggests a higher population prevalence than in previous literature (26.5% in 2006), with increasing prevalence within each age group as individuals aged. Our overall crude incidence rate of 47.1 per 1,000 PYAR is consistent with previous pooled estimates of 43.4 per 1,000 PYAR. The finding that at least one in 10 people aged 50–64 are already frail is noteworthy, as is the scale of change in prevalence within the study period, with significant increases in moderate to severe frailty in all age groups, tripling in the 50–64 age group and approximately doubling in all others. The estimated incidence in the younger age group was also higher than expected, at 31.8 per 1,000 PYAR.

The multi-state model demonstrated an increasing speed of transitions with age, consistent with studies predominantly using phenotypic frailty assessments [21, 35]. Within each age group, the longer time spent within severe frailty may be explained by a saturation effect of deficits for each individual, with no further frailty state transitions other than death possible. The model established that in addition to recognised risk factors for frailty (age and female sex), deprivation, Asian ethnicity and urban residence were independently associated with an increased risk of frailty transitions in all groups. This analysis shows that socio-economic factors such as deprivation, ethnicity and urban residence have a significant impact on frailty. Deprivation was the most important factor after age, with people living in the two most deprived IMD quintiles having earlier onset of frailty and faster progression. This aligns with previous studies suggesting that older people with greater socioeconomic deprivation spend longer in frail states [36–42].

Early onset in deprived groups, combined with the length of time that people spend in the severely frail state, suggests a long period of need associated with frailty and the importance of prevention across the life course to address inequity in frailty burden. Higher frailty onset and progression in people of Asian ethnicity explain differences in the prevalence of frailty with ethnicity observed in a London cohort [10], and suggest that tailored approaches for different communities may be important. The higher transition rate in people living in urban areas indicates that geographical considerations might also be important, in line with recent results from a small English cohort which suggested that coastal communities might be at higher risk [43]. Overall, the independent associations identified in this analysis indicate that targeted prevention, and intervention to slow progression, could be beneficial in reducing population impact of frailty. However, there is still limited evidence for clinical guidelines for preventing, delaying or reversing frailty, apart from in specific contexts. Therefore significant investment in research to identify the most effective preventative strategies that enable people to remain in fit or mild frailty states is paramount [21, 44, 45].

These findings are likely to be of particular importance when planning services, given that most frailty services are currently targeted at those aged 65 and above. Although high prevalence of severe frailty in older age groups is the focus of considerable policy and practice attention, our analyses demonstrate that absolute numbers of younger old people with mild and moderate frailty exceed those of older people with severe frailty. This suggests that population-level preventative strategies are needed and could have more impact than a focus on severe frailty. It is also important to note that our analysis demonstrates that older age groups transition to higher levels of frailty faster than middle-aged adults. A population-level approach to prevention of frailty or slowing of frailty progression earlier in the life course is therefore likely to be a key strategy for long-term reduction of population morbidity, disability and service use. Further work is necessary to understand points in the frailty trajectory where intervention will have most impact at population level. Although it has been shown that people with more severe frailty have higher healthcare costs [46], analysis of the current and future population impact of frailty in terms of service use and costs is essential and is the focus of ongoing work within this project [46].

## Limitations

This analysis suggests a higher prevalence in people aged 50–64 than reported elsewhere utilising different frailty indices and data sources [43], but otherwise patterns of frailty onset and progression are consistent with overall trends. Deficits-based FIs produce higher overall frailty prevalence estimates than phenotypic scores, but give better discrimination in patients with mild frailty, which is related to poorer outcomes and therefore useful for service planning [47].

The calculation of the eFI score depends on the quality and completeness of the EHR data, which can be influenced by policy and practice conventions. Increasing prevalence could have been driven by more complete coding of eFI deficits, although methods of calculating the eFI in English general practice were introduced in 2016, so would be unlikely to have affected coding during the study period. Similarly, Quality and Outcomes Framework coding, introduced during this time period, might have increased recording of diseases that contribute to the eFI score. However, no sharp changes in prevalence that might suggest coding impact were observed. The use of the most recent address in identifying urban/rural location could mask selective migration between residential areas and types driven by frailty status.

The eFI is a cumulative measure based on conditions recorded in the EHR; although reversals are possible, in clinical practice it is uncommon for clinicians to remove codes from the record, other than for medications (polypharmacy). For this reason, we found few improvements in frailty status over time. The eFI has, however, been shown to demonstrate progression in frailty in longitudinal studies [22, 23], a property required to achieve the aims of this study.

Ethnicity data were incomplete within primary care, so were supplemented with linked secondary care data. The two data sources used different ethnicity categories, necessitating broad aggregation of ethnicity groups. Categories were further collapsed for the MSM due to computational demand. Despite this, the model demonstrated that ethnicity was an independent factor in transitions, suggesting that this is an area worthy of more detailed study.

## Conclusions

This study is unique in exploring frailty transitions in an ageing population, including people aged ≥50, and demonstrates frailty is already prevalent before age 65. It provides new evidence on the rate of decline within an ageing cohort, demonstrating frailty transitions were independently associated with both older age and socioeconomic factors. We provide new evidence about high prevalence of frailty within the ageing population and the social inequity in patterns of frailty. Slower transition rates in middle age and earlier onset in some groups might present an opportunity to reduce health disparities through for earlier identification and multisectoral intervention to slow progression and reduce care needs.

## Supplementary Material

aa-22-1458-File002_afad058

## Data Availability

The data that support the findings of this study are available from the RCGP, RSC and NHS Digital, but restrictions apply to the availability of these data, which were used following approvals and data sharing agreements for the current study, and so are not publicly available.
