# Supplementary material for: Frailty transitions and prevalence in an ageing population: longitudinal analysis of primary care data from an open cohort of adults aged 50 and over in England, 2006–2017
Source: Age Ageing. 2023 May 2;52(5):afad058. doi: 10.1093/ageing/afad058 (PMC10158172; doi:10.1093/ageing/afad058)
Supplement: aa-22-1458-File002_afad058 [file aa-22-1458-file002_afad058.docx]

**Frailty transitions and prevalence in an ageing population: longitudinal analysis of primary care data from an open cohort of adults aged 50 and over in England, 2006-2017**

**Appendix 1 Multi-state model structure**


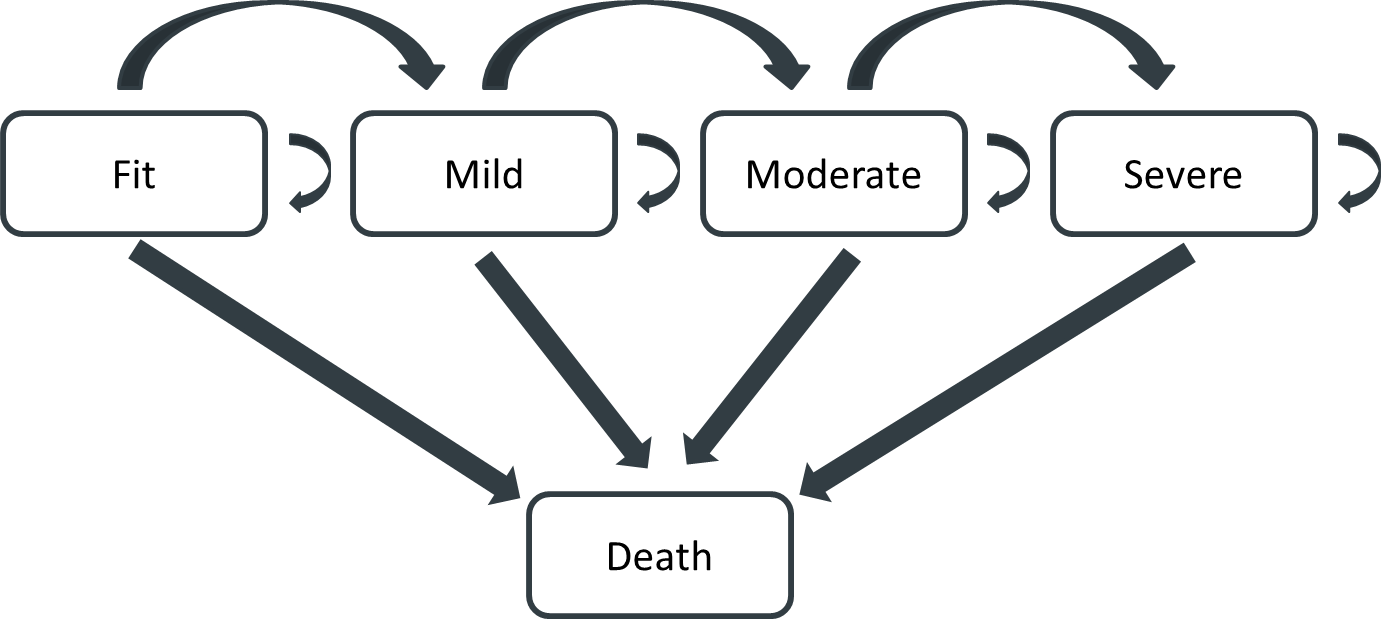


**Appendix 2 Incidence rates by age group by calendar year**

|  | **Age group** | | | |
| --- | --- | --- | --- | --- |
| **Calendar year** | **50-64** | **65-74** | **75-84** | **≥85** |
| **2007** | 28.6  [28.1 – 29.0] | 84.8  [83.5 – 86.1] | 165.3  [162.7 – 167.9] | 217.6  [211.9 – 223.5] |
| **2008** | 27.1  [26.7 – 27.5] | 76.4  [75.2 – 77.6] | 148.8  [146.3 – 151.4] | 201.9  [196.0 – 207.9] |
| **2009** | 27.2  [26.8 – 27.6] | 74.0  [72.8 – 75.2] | 138.0  [135.4 – 140.5] | 194.5  [188.5 – 200.6] |
| **2010** | 27.5  [27.1 – 28.0] | 74.1  [72.9 – 75.3] | 138.0  [135.0 – 140.2] | 191.0  [184.8 – 197.3] |
| **2011** | 27.1  [26.7 – 27.5] | 70.4  [69.3 – 71.6] | 131.9  [129.4 – 134.5] | 183.4  [177.2 – 189.8] |
| **2012** | 26.8  [26.4 – 27.3] | 67.1  [66.0 – 68.3] | 126.0  [123.5 – 128.6] | 174.8  [168.7 – 181.2] |
| **2013** | 27.7  [27.3 – 28.2] | 67.3  [66.2 – 68.4] | 126.7  [124.2 – 129.3] | 183.1  [176.7 – 189.7] |
| **2014** | 28.4  [28.0 – 28.8] | 67.6  [64.6 – 66.7] | 129.9  [127.3 – 132.5] | 195.7  [189.0 – 202.7] |
| **2015** | 27.6  [27.2 – 28.0] | 65.6  [64.6 – 66.7] | 127.3  [124.8 – 129.9] | 193.4  [186.6 – 200.5] |
| **2016** | 27.3  [26.9 – 27.7] | 65.3  [64.3 – 66.4] | 130.6  [128.0 – 133.3] | 195.5  [188.6 – 202.7] |
| **2017** | 28.5  [28.1 – 29.0] | 65.8  [64.7 – 66.8] | 128.3  [125.8 – 131.0] | 199.0  [191.9 – 206.5] |

**Appendix 3 Total number and percentage of people within each frailty category 2006-2017 by age group**

| **Calendar year** | **Age group 50-64** | | | | | | | | |
| --- | --- | --- | --- | --- | --- | --- | --- | --- | --- |
|  | **Fit (n, %)** | | **Mild (n, %)** | | **Moderate (n, %)** | | **Severe (n, %)** | | **Total** |
| 2006 | 516468 | 89.2% | 56806 | 9.8% | 5397 | 0.9% | 528 | 0.1% | 579199 |
| 2007 | 526828 | 87.9% | 64958 | 10.8% | 6833 | 1.1% | 710 | 0.1% | 599329 |
| 2008 | 534114 | 86.8% | 72112 | 11.7% | 8077 | 1.3% | 891 | 0.1% | 615194 |
| 2009 | 539746 | 85.8% | 78808 | 12.5% | 9366 | 1.5% | 1057 | 0.2% | 628977 |
| 2010 | 543754 | 84.9% | 85046 | 13.3% | 10793 | 1.7% | 1240 | 0.2% | 640833 |
| 2011 | 553787 | 84.0% | 91806 | 13.9% | 12217 | 1.9% | 1453 | 0.2% | 659263 |
| 2012 | 557393 | 83.3% | 96358 | 14.4% | 13310 | 2.0% | 1682 | 0.3% | 668743 |
| 2013 | 564539 | 82.8% | 101212 | 14.8% | 14422 | 2.1% | 1913 | 0.3% | 682086 |
| 2014 | 572356 | 82.1% | 106641 | 15.3% | 15817 | 2.3% | 2149 | 0.3% | 696963 |
| 2015 | 585640 | 81.6% | 112484 | 15.7% | 17215 | 2.4% | 2490 | 0.3% | 717829 |
| 2016 | 597759 | 81.1% | 118080 | 16.0% | 18533 | 2.5% | 2807 | 0.4% | 737179 |
| 2017 | 605582 | 80.4% | 124330 | 16.5% | 20429 | 2.7% | 3218 | 0.4% | 753559 |
|  | **Age group 65-74** | | | | | | | | |
|  | **Fit (n, %)** | | **Mild (n, %)** | | **Moderate (n, %)** | | **Severe (n, %)** | | **Total** |
| 2006 | 187162 | 69.4% | 69189 | 25.6% | 11979 | 4.4% | 1462 | 0.5% | 269792 |
| 2007 | 182478 | 66.4% | 75536 | 27.5% | 14785 | 5.4% | 2102 | 0.8% | 274901 |
| 2008 | 181870 | 64.4% | 80838 | 28.6% | 17219 | 6.1% | 2602 | 0.9% | 282529 |
| 2009 | 183204 | 62.6% | 86749 | 29.7% | 19314 | 6.6% | 3169 | 1.1% | 292436 |
| 2010 | 187369 | 61.3% | 93107 | 30.5% | 21575 | 7.1% | 3658 | 1.2% | 305709 |
| 2011 | 188626 | 60.1% | 97515 | 31.1% | 23654 | 7.5% | 4127 | 1.3% | 313922 |
| 2012 | 197398 | 59.6% | 103626 | 31.3% | 25616 | 7.7% | 4682 | 1.4% | 331322 |
| 2013 | 208023 | 59.1% | 110766 | 31.5% | 28097 | 8.0% | 5242 | 1.5% | 352128 |
| 2014 | 213851 | 58.4% | 116015 | 31.7% | 30110 | 8.2% | 5974 | 1.6% | 365950 |
| 2015 | 219306 | 57.8% | 121458 | 32.0% | 32260 | 8.5% | 6712 | 1.8% | 379736 |
| 2016 | 223302 | 57.1% | 125816 | 32.2% | 34489 | 8.8% | 7570 | 1.9% | 391177 |
| 2017 | 224938 | 56.2% | 129731 | 32.4% | 37020 | 9.3% | 8427 | 2.1% | 400116 |
|  | **Age group 75-84** | | | | | | | | |
|  | **Fit (n, %)** | | **Mild (n, %)** | | **Moderate (n, %)** | | **Severe (n, %)** | | **Total** |
| 2006 | 86398 | 46.2% | 71846 | 38.5% | 23589 | 12.6% | 4979 | 2.7% | 186812 |
| 2007 | 79804 | 42.0% | 75347 | 39.6% | 28262 | 14.9% | 6820 | 3.6% | 190233 |
| 2008 | 75712 | 38.9% | 78478 | 40.4% | 31950 | 16.4% | 8247 | 4.2% | 194387 |
| 2009 | 72473 | 36.8% | 80114 | 40.7% | 34828 | 17.7% | 9534 | 4.8% | 196949 |
| 2010 | 69897 | 34.8% | 82174 | 40.9% | 38014 | 18.9% | 10986 | 5.5% | 201071 |
| 2011 | 68526 | 33.3% | 84539 | 41.0% | 40672 | 19.7% | 12261 | 6.0% | 205998 |
| 2012 | 68123 | 32.3% | 86253 | 40.9% | 43047 | 20.4% | 13672 | 6.5% | 211095 |
| 2013 | 68009 | 31.3% | 88380 | 40.7% | 45548 | 21.0% | 15282 | 7.0% | 217219 |
| 2014 | 67691 | 30.5% | 89981 | 40.5% | 47724 | 21.5% | 16904 | 7.6% | 222300 |
| 2015 | 67962 | 29.8% | 91430 | 40.1% | 49864 | 21.9% | 18697 | 8.2% | 227953 |
| 2016 | 67487 | 29.2% | 91509 | 39.6% | 51594 | 22.3% | 20295 | 8.8% | 230885 |
| 2017 | 66815 | 28.7% | 91321 | 39.2% | 52705 | 22.6% | 22030 | 9.5% | 232871 |
|  | **Age group 85 and above** | | | | | | | | |
|  | **Fit (n, %)** | | **Mild (n, %)** | | **Moderate (n, %)** | | **Severe (n, %)** | | **Total** |
| 2006 | 21356 | 31.3% | 27977 | 40.9% | 14354 | 21.0% | 4645 | 6.8% | 68332 |
| 2007 | 19596 | 26.8% | 29372 | 40.2% | 17621 | 24.1% | 6531 | 8.9% | 73120 |
| 2008 | 18239 | 23.7% | 30401 | 39.6% | 20027 | 26.1% | 8178 | 10.6% | 76845 |
| 2009 | 17121 | 21.3% | 31323 | 38.9% | 22428 | 27.9% | 9555 | 11.9% | 80427 |
| 2010 | 16042 | 19.1% | 31647 | 37.7% | 24926 | 29.7% | 11378 | 13.5% | 83993 |
| 2011 | 15368 | 17.6% | 32204 | 36.8% | 26786 | 30.6% | 13206 | 15.1% | 87564 |
| 2012 | 14946 | 16.3% | 32549 | 35.5% | 28957 | 31.6% | 15203 | 16.6% | 91655 |
| 2013 | 14387 | 15.2% | 32900 | 34.8% | 30369 | 32.1% | 16838 | 17.8% | 94494 |
| 2014 | 13681 | 14.1% | 32855 | 33.9% | 31716 | 32.8% | 18555 | 19.2% | 96807 |
| 2015 | 13257 | 13.3% | 32692 | 32.8% | 33036 | 33.1% | 20772 | 20.8% | 99757 |
| 2016 | 12964 | 12.7% | 32206 | 31.5% | 33987 | 33.3% | 22989 | 22.5% | 102146 |
| 2017 | 12332 | 12.0% | 31181 | 30.3% | 34265 | 33.3% | 25171 | 24.4% | 102949 |
